# Eating attitudes and behaviours in a sample of female university students: does studying nutrition and dietetics make a difference?

**DOI:** 10.1186/2050-2974-2-S1-P12

**Published:** 2014-11-24

**Authors:** Tetyana Rocks, Fiona Pelly, Lisa Martin, Gary Slater

**Affiliations:** 1 University of the Sunshine Coast, Sunshine Coast, Australia

## 

Eating attitudes and behaviours of female students enrolled in the discipline of Nutrition and Dietetics (ND) in Australia has not been previously explored. Therefore, the aim of this study was to examine these characteristics in undergraduate ND students in comparison to a group of female students enrolled in Occupational Therapy (OT). A cross-sectional data was initiated in August-October 2013 as part of longitudinal research investigating dietary and exercise behaviours and practices, plus body composition of this cohort. Previously validated questionnaires, including the Eating Attitudes Test, the Three-Factor Eating Questionnaire, the Rosenberg Self-Esteem Scale, and the Body Shape Questionnaire were administered to students to assess eating behaviours, self-esteem and body dissatisfaction respectively. Overall, 119 females students (75 ND and 44 OT) participated in this part of the study. Preliminary results suggest no significant differences in eating behaviours, self-esteem and body dissatisfaction between student populations. However, almost two thirds of the participants have indicated mild to marked concern with body shape despite the mean reported Body Mass Index of 23.1kg/m2. The associations between eating attitudes and demographic, physiological and psychological characteristics of this sample will be presented. Implications for future studies in this population will be discussed.

